# A systems-oriented Ayurvedic approach in chronic plaque psoriasis with renal inflammatory indicators: a 2-year longitudinal case report

**DOI:** 10.3389/fmed.2026.1882948

**Published:** 2026-07-06

**Authors:** Sreelekshmi Mallika, Arathy Menon, Vishnu Unnikrishnan, Preethi Mohan

**Affiliations:** 1 Department of Agadatantra, Amrita School of Ayurveda, Amrita Vishwa Vidyapeetham, Kollam, India; 2 Department of Kayachikitsa, Shree Swaminarayan Ayurvedic College, Swaminarayan University, Gandhinagar, Gujarat, India

**Keywords:** Ayurveda, chronic plaque psoriasis, IgA nephropathy, integrative medicine, longitudinal follow-up, PASI, psoriasis, psychoneuroimmunology

## Abstract

**Background:**

Psoriasis is increasingly recognized as a chronic systemic inflammatory disorder associated with multisystem involvement, including psychological and renal manifestations. Emerging evidence suggests possible links between psoriasis, inflammatory renal abnormalities, psychoneuroimmunological stress pathways, and metabolic dysregulation. However, longitudinal integrative management data with extended follow-up remain limited.

**Case presentation:**

A 22-year-old female with chronic plaque psoriasis of 4 years duration and childhood nephrotic syndrome with prolonged corticosteroid exposure presented with extensive erythematous scaly plaques associated with severe itching, burning sensation, and recurrent relapses. Baseline evaluation demonstrated positive candle grease sign and Auspitz sign with a Psoriasis Area and Severity Index (PASI) score of 52. Laboratory investigations revealed persistent urinary abnormalities, including elevated urine protein–creatinine ratio (UPCR), microscopic hematuria, and trace albuminuria despite preserved renal function. Perceived Stress Scale (PSS) scores improved from 37 at baseline to 25 following the first treatment schedule, increased to 28 during recurrence, and subsequently decreased to 17 after the second treatment schedule. The patient underwent a staged Ayurvedic treatment protocol consisting of Deepana–Pachana, Snehapana, Abhyanga, Swedana, Vamana, Takradhara, and follow-up medications. Marked clinical improvement was observed following treatment, with PASI score reducing from 52 to 6, improvement in stress scores, and reduction in urinary inflammatory parameters. The patient subsequently maintained near-complete remission for approximately 2 years. Mild recurrence associated with academic stress occurred in 2026 with PASI score of 12 and absence of severe inflammatory signs previously observed during baseline presentation. A second treatment schedule incorporating Deepana–Pachana, Snehapana, Virechana, and Takradhara again resulted in substantial improvement, with PASI score reducing to 5 and further improvement in urinary findings and psychological assessment scores.

**Conclusion:**

This case highlights the potential role of staged integrative Ayurvedic intervention in achieving sustained remission and reducing inflammatory severity in chronic plaque psoriasis associated with psychological stress and persistent urinary abnormalities. Parallel improvement in dermatological severity, stress assessment, digestive-metabolic status, and urinary parameters suggests possible systemic effects involving interconnected inflammatory and psychoneuroimmunological pathways. Further large-scale longitudinal studies integrating clinical, biochemical, immunological, and psychometric assessment are required to validate these observations.

## 1 Introduction

Psoriasis is a chronic immune-mediated inflammatory disorder increasingly recognized as a systemic disease rather than a condition confined to the skin (1). Dysregulation of inflammatory pathways involving the Th1/Th17 axis and cytokine networks contributes to persistent systemic inflammation and keratinocyte hyperproliferation (2). Emerging evidence from immunogenetic studies further suggests that psoriasis represents a biologically heterogeneous disorder involving interconnected inflammatory, metabolic, and genetic pathways, thereby supporting the concept of individualized disease expression and therapeutic response (3).

Beyond its dermatological manifestations, psoriasis has been associated with multiple systemic comorbidities, including cardiovascular, metabolic, arthritic, and renal involvement (4). Increasing evidence suggests that both overt and subclinical renal abnormalities may occur in psoriasis, even in the absence of clinically apparent kidney dysfunction (5). Recent clinicopathological studies have demonstrated possible associations between psoriasis and IgA nephropathy, suggesting overlapping immune-inflammatory mechanisms between cutaneous and renal disease (6). Furthermore, genetic and transcriptomic analyses have identified potential biological links between psoriasis and IgA nephropathy, supporting the concept of shared inflammatory pathways extending beyond the skin (7).

Psychological stress is recognized as an important trigger in psoriasis and is increasingly understood to influence disease activity through psychoneuroimmunological pathways. Bidirectional interactions between inflammatory signaling, neuroendocrine responses, and psychological well-being may contribute to both disease exacerbation and impaired quality of life (8). Contemporary studies have demonstrated significant associations between psoriasis severity, anxiety, depression, stress burden, and psychosocial impairment, highlighting the importance of incorporating psychological assessment into long-term disease management (9).

Despite significant advances in psoriasis therapeutics, including topical agents, systemic immunomodulators, biologics, and targeted anti-inflammatory therapies, long-term management remains challenging because of recurrent relapses, prolonged treatment requirements, variable therapeutic response, and potential adverse effects associated with chronic corticosteroid or immunosuppressive use (10). Increasing emphasis has therefore been placed on individualized and systems-oriented therapeutic strategies capable of addressing the multifactorial nature of chronic inflammatory disease.

Emerging evidence also suggests important interactions between metabolism, the microbiome, and immune regulation in psoriasis pathogenesis. Integrated microbiome–metabolome analyses have demonstrated microbial–metabolic interactions contributing to inflammatory signaling and systemic disease activity in psoriasis (11). From an Ayurvedic perspective, psoriasis-like conditions are understood as disorders involving impairment of Agni, broadly representing digestive-metabolic regulatory function, resulting in systemic imbalance and tissue pathology. Ayurvedic management approaches emphasizing restoration of metabolic regulation followed by individualized purification-based interventions (Shodhana) have increasingly gained attention as potential integrative approaches for chronic inflammatory disorders, although high-quality evidence remains limited (12).

The present report describes a young female with chronic plaque psoriasis, persistent urinary abnormalities, significant psychological stress, and a history of prolonged corticosteroid exposure who demonstrated sustained remission and reduction in urinary inflammatory markers following staged Ayurvedic intervention over a two-year follow-up period. The case highlights the potential interplay between systemic inflammation, renal involvement, metabolic regulation, and psychoneuroimmunological factors in psoriasis, while also emphasizing the need for further integrative research incorporating clinical, biochemical, immunological, and longitudinal outcome assessment.

## 2 Case presentation

### 2.1 Patient information

A 22-year-old female college student presented with chronic plaque psoriasis of 4 years duration. The patient had a history of childhood nephrotic syndrome diagnosed at the age of 6 years and had received corticosteroid therapy for approximately 16 years, primarily for the management of recurrent nephrotic syndrome relapses. Plaque psoriasis developed a few months after withdrawal of corticosteroid treatment and followed a chronic relapsing course over the subsequent 4 years. Following the onset of psoriasis in 2020, the patient received corticosteroid-based treatment for approximately 4 years. Clinical symptoms remained controlled during corticosteroid use; however, recurrent flare-ups occurred following discontinuation of therapy, resulting in a chronic relapsing disease course. No additional systemic immunosuppressive agents, biologics, or disease-modifying therapies were used during this period. There was no history of diabetes mellitus, hypertension, or other major systemic comorbidities. Family history of psoriasis was absent.

The patient initially developed plaque-like skin lesions at the age of 18 years, which progressively increased in severity and extent over time. She had been receiving corticosteroid therapy for psoriasis over the preceding 4 years; however, recurrent exacerbations occurred following discontinuation of medication. The patient reported significant psychosocial distress related to the visibility of skin lesions, including social withdrawal, reduced self-confidence, and difficulty in interpersonal interactions. Stress, particularly related to academic activities and examinations, was identified as a major aggravating factor.

The patient also reported reduced appetite, while bowel habits and sleep were largely normal. Symptoms were aggravated during hot climatic conditions. Owing to the chronic relapsing nature of the disease and inadequate long-term control with previous treatment, she sought Ayurvedic management.

### 2.2 Clinical findings

On presentation, the patient presented with extensive erythematous scaly plaques associated with severe itching, burning sensation, and recurrent episodes of excoriation with minor bleeding following scratching. No clinical evidence of secondary skin infection was observed during either treatment episode. Routine hematological investigations were within normal limits.

The lesions demonstrated progressive spread with recurrent exacerbations over the preceding years. Psychological stress was consistently associated with worsening of symptoms. At the time of recurrence during follow-up in 2026, lesions were significantly milder in comparison to the initial presentation and consisted of a few small plaques over both lower limbs and a minimal lesion over the right elbow without associated itching or discomfort (Table 1).

**Table 1:** Baseline clinical and laboratory findings.

Parameter	Findings
Age/Sex	22-year-old female
Occupation	College student
Duration of psoriasis	4 years
Past medical history	Childhood nephrotic syndrome
Corticosteroid exposure	Approximately 16 years
Presenting symptoms	Itching, burning sensation, scaling, recurrent plaques
Distribution of lesions	Multiple body regions including scalp and lower limbs
Trigger factors	Psychological stress, hot climate
Candle grease sign	Positive
Auspitz sign	Positive
PASI score	52
PSS score	37
JAQ score	5
UPCR	52
Urine RBC/hpf	6–7
Albuminuria	Trace albumin present
Urine culture	*Enterococcus faecalis* positive
Serum IgA	0.74 g/L
Renal function	Normal serum creatinine and eGFR

### 2.3 Diagnostic assessment

Clinical diagnosis of chronic plaque psoriasis was established based on characteristic morphology and distribution of lesions. During the initial presentation in 2024, classical dermatological signs including positive candle grease sign and Auspitz sign were elicited. The lesions were characterized by erythematous plaques with scaling and associated inflammatory changes involving multiple body regions including the scalp.

At the time of recurrence in 2026, the lesions were considerably milder in comparison to the initial presentation. Notably, candle grease sign and Auspitz sign were absent during the second presentation, suggesting reduction in inflammatory severity relative to the earlier disease phase.

Laboratory investigations performed on 13/02/2024 demonstrated microscopic hematuria with 6–7 red blood cells/high-power field and 3–4 pus cells/high-power field on urine microscopy. Urine appeared reddish in color. Urine culture revealed growth of *Enterococcus faecalis*, although the patient did not report symptoms suggestive of urinary tract infection.

The urine protein–creatinine ratio (UPCR) was elevated at 52, and urine routine examination demonstrated trace albumin. Serum IgA levels were at the lower limit of the reference range (0.74 g/L). Renal function parameters, including serum creatinine and estimated glomerular filtration rate (eGFR), were within normal limits, and no clinical evidence of edema or fluid overload was present. Urine cultures demonstrated asymptomatic bacteriuria with growth of *Enterococcus faecalis* during the initial presentation and *Escherichia coli* during recurrence evaluation. These findings may represent potential confounding factors while interpreting urinary inflammatory parameters and serum IgA variation.

Psychological assessment using the Perceived Stress Scale (PSS) revealed a baseline score of 37, indicating significant stress burden. Assessment of digestive-metabolic status using the Jatharagni Assessment Questionnaire (JAQ) demonstrated impaired Agni function with a baseline score of 5.

Repeat investigations performed on 24/04/2024 after completion of the first treatment schedule demonstrated reduction of UPCR from 52 to 42, absence of detectable albumin, negative urine culture, and clear urine appearance. Microscopic hematuria persisted with 3–4 red blood cells/high-power field.

At recurrence in April 2026, laboratory evaluation demonstrated further improvement, with UPCR reduced to 20 and urine microscopy showing 2–3 red blood cells/high-power field without albuminuria.

Renal biopsy and advanced immunological investigations were not performed. Therefore, although persistent urinary abnormalities suggested possible renal inflammatory involvement, definitive characterization of renal pathology could not be established.

### 2.4 Therapeutic intervention

#### 2.4.1 First treatment schedule (2024)

**Figure 1:**
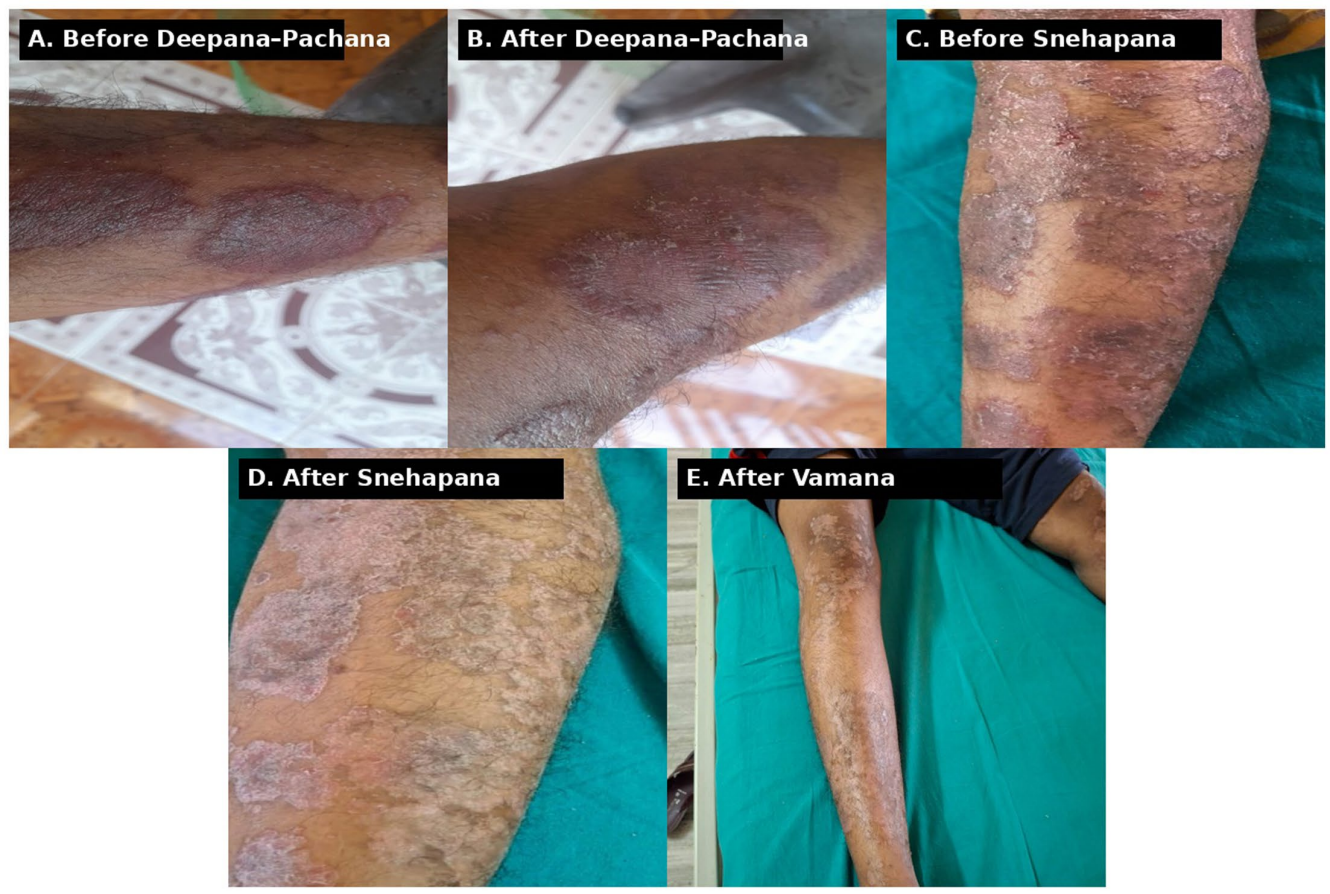
Sequential clinical progression during the first treatment schedule. **(A)** Baseline presentation before initiation of Deepana–Pachana demonstrating extensive erythematous psoriatic plaques with scaling and active inflammatory changes. **(B)** Partial reduction in inflammatory activity after Deepana–Pachana therapy. **(C)** Clinical appearance after Snehapana showing reduction in active inflammatory changes with persistent scaling and plaque formation. **(D)** Marked reduction in erythema and inflammatory activity after Vamana with residual scaling and post-inflammatory hyperpigmentation. **(E)** Clinical appearance after Vamana demonstrating marked reduction in erythema and inflammatory activity with residual post-inflammatory hyperpigmentation.

During the first treatment schedule, Deepana–Pachana was performed using Panchakola Choorna, Khadirarishta, Patolamooladi Kashaya, and Guggulu Panchapala Choorna. Snehapana was administered with Aragvadha Mahatiktaka Ghrita in escalating doses. Following Abhyanga with Lantthapala Kera Taila combined with Aragvadha Mahatiktaka Ghrita and Swedana using Aragvadhadi Gana, Vamana was performed using a Madanaphala-based emetic formulation. Takradhara was subsequently administered using medicated buttermilk processed with Aragvadhadi Gana (Figure 1).

##### 2.4.1.1 Phase 1: Deepana–Pachana therapy (digestive and metabolic corrective therapy)

The patient was initially managed on an outpatient basis with Deepana–Pachana therapy aimed at improving digestive and metabolic function (Agni) prior to purification procedures. Herbal decoctions and Deepana–Pachana medications were administered for approximately 1 months. Clinical appearance after Deepana–Pachana therapy demonstrating a relatively drier appearance of plaques with improvement in subjective symptoms including pruritus and burning sensation prior to subsequent interventions.

##### 2.4.1.2 Phase 2: Snehapana (therapeutic internal oleation)

Subsequently, the patient was admitted for inpatient management and underwent Snehapana (internal oleation) for 7 days. Progressive drying of lesions was noted during the procedure, and by the seventh day, lesions had reduced to predominantly dry hypopigmented patches.

##### 2.4.1.3 Phase 3: Abhyanga and Swedana (external oleation and sudation therapy)

Following Snehapana, Abhyanga (external oleation) and Swedana (sudation therapy) were administered for one day as preparatory procedures.

##### 2.4.1.4 Phase 4: Vamana (therapeutic emesis)

Vamana was subsequently performed as the primary purification procedure. The patient was monitored post-procedure with one day of rest.

##### 2.4.1.5 Phase 5: Takradhara and Thalam

(Pouring of medicated buttermilk over the forehead and Application of medicated oil and herbal powder over the scalp)

Takradhara was then administered for 7 consecutive days. The patient was discharged with appropriate internal medications and advised regular follow-up.

Antimicrobial formulations, along with increased hydration, were concurrently administered for approximately 1.5 months in view of the positive urine culture findings.

At one-month follow-up, near-complete resolution of psoriatic lesions was observed, with marked improvement in skin appearance and resolution of post-inflammatory hypopigmented lesions over the face and neck. PSS score improved from 37 to 25, while JAQ score increased from 5 to 11 following treatment. Culture result also turned negative.

#### 2.4.2 Second treatment schedule (2026)

The second treatment schedule was modified in comparison with the initial intervention because the recurrence was clinically less severe than the baseline presentation (PASI 12 vs. 52). Accordingly, Virechana was selected instead of Vamana as the principal Shodhana procedure.

After approximately 2 years of sustained remission, the patient returned on 02/04/2026 with mild recurrence of lesions associated with increased academic stress. Baseline PSS score at recurrence was 28, while JAQ score was 8.

##### 2.4.2.1 Phase 1: Deepana–Pachana therapy (digestive-metabolic corrective therapy)

Deepana–Pachana therapy was administered for 2 weeks prior to purification procedures.

##### 2.4.2.2 Phase 2: Snehapana (therapeutic internal oleation)

The patient was admitted on 10/04/2026 and underwent Snehapana for 7 days.

##### 2.4.2.3 Phase 3: Abhyanga and Swedana (external oleation and sudation therapy)

This was followed by Abhyanga and Swedana for 3 days.

##### 2.4.2.4 Phase 4: Virechana (therapeutic purgation)

Virechana was then performed as the principal purification procedure during the second treatment schedule.

##### 2.4.2.5 Phase 5: Takradhara and Thalam

(Pouring of medicated buttermilk over the forehead and Application of medicated oil and herbal powder over the scalp)

**Figure 2:**
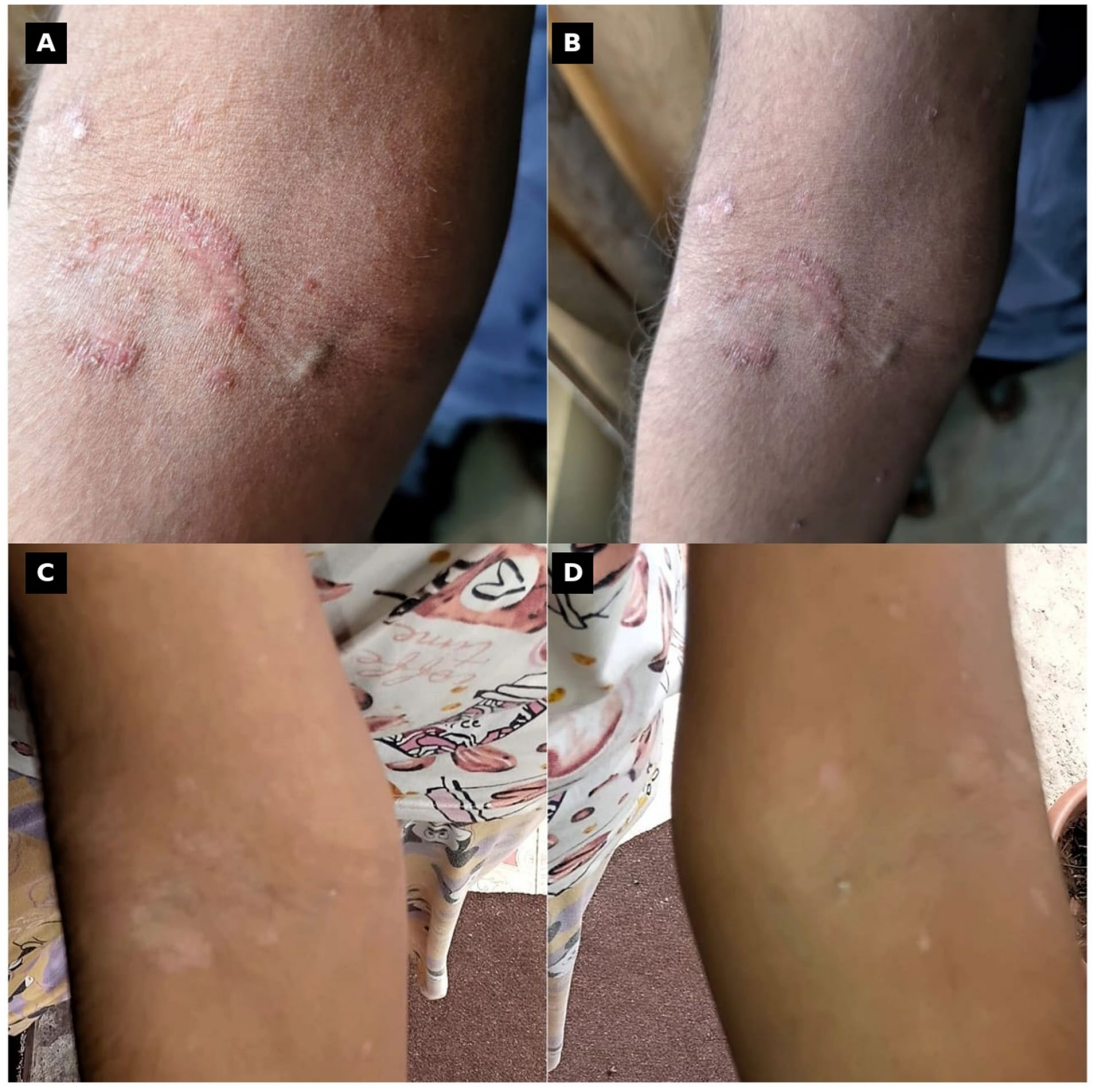
Clinical progression during the second treatment schedule. **(A)** Clinical appearance at the time of mild recurrence prior to initiation of the second treatment schedule. **(B)** Early response following commencement of the second treatment schedule, showing reduction in scaling and inflammatory activity. **(C)** Further clinical improvement with marked reduction in lesion size and severity. **(D)** Near-complete resolution of residual lesions with minimal post-inflammatory changes following completion of the second treatment schedule.

**Figure 3:**
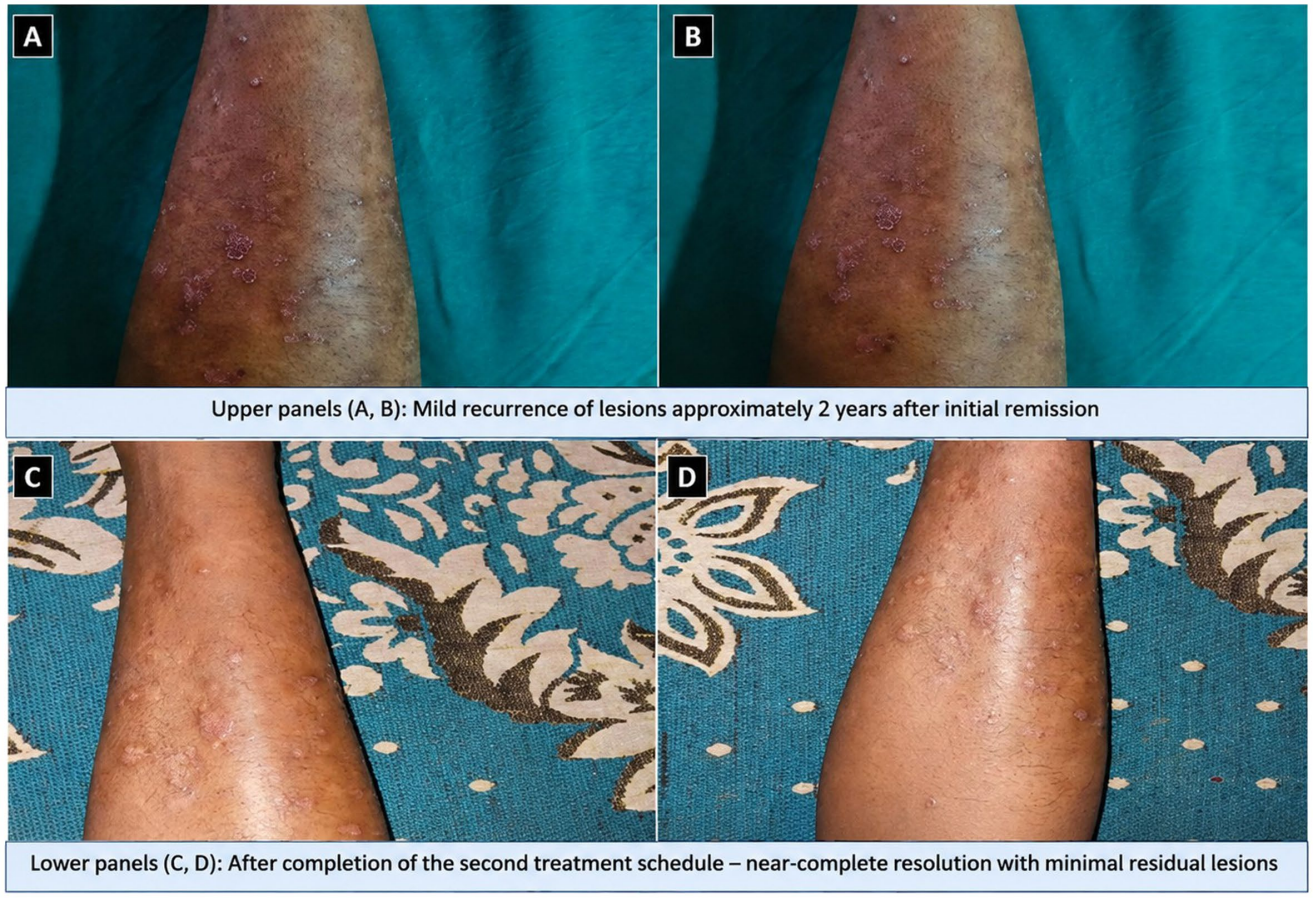
Clinical appearance during mild recurrence approximately two years after initial remission and after completion of the second treatment schedule. **(A, B)** Clinical appearance during mild recurrence approximately two years after initial remission, showing limited residual psoriatic activity. **(C, D)** Clinical appearance after completion of the second treatment schedule, demonstrating reduced inflammatory severity and near-complete resolution of residual lesions.

Post-procedure Takradhara was administered for 7 days (Figures 2, 3).

The clinical photographs shown are representative lesions and do not reflect the complete body surface area involvement considered during PASI assessment.

Following treatment, marked improvement in lesions was again observed, with only minimal residual marks remaining. PSS score improved from 28 to 17, while JAQ score increased from 8 to 12. Follow-up laboratory evaluation demonstrated further reduction in UPCR and improvement in urinary findings. During the second treatment schedule, antimicrobial formulations were continued for the one-month follow-up period due to recurrent asymptomatic bacteriuria. Follow-up urine culture results were pending at the time of manuscript preparation (Table 2).

**Table 2:** Detailed therapeutic schedule during the two treatment phases.

Treatment phase	Intervention	Formulation/Procedure	Dose/Duration	Purpose
First treatment schedule (2024)				
Phase 1	Deepana–Pachana	Khadirarishtam	25 mL twice daily after food	Deepana–Pachana and reduction of inflammatory activity
		Panchakola Choornam	5 g with arishta twice daily	Agni improvement and metabolic correction
		Guggulu Panchapalam Choornam	10 g with hot water twice daily after food	Reduction of inflammation and metabolic regulation
		Patolamooladi Kashayam	15 mL mixed with 45 mL water twice daily before food	Reduction of inflammatory manifestations
Phase 2	Snehapana	Aragvadha Mahathikthakam Ghritham	Increasing dose from 50 mL to 220 mL over 7 days	Internal oleation prior to Shodhana
Phase 3	Abhyanga	Lanthapala Kera Tailam mixed with Aragvadha Mahathikthakam Ghritham	1 day	External oleation
	Swedana	Aragvadhadi Gana	1 day	Sudation therapy prior to Vamana
Phase 4	Vamana	Madanaphala Yoga	Single procedure; 10–12 vegas observed	Principal Shodhana procedure
Phase 5	Takradhara	Aragvadhadi Gana processed medicated buttermilk	7 days	Stress reduction and psychological stabilization
	Thalam	Ksheerabala Tailam with Kachooradi Choornam	During Takradhara period	Mental relaxation and calming effect
Discharge medication	Internal medication	Gugguluthikthakam Ghritham	1 tablespoon early morning on empty stomach	Maintenance therapy
	Internal medication	Kaishora Guggulu Gulika	2–0-2	Long-term inflammatory regulation
	External application	Lanthapala Kera Tailam	Local application	Maintenance of skin health
Second treatment schedule (2026)				
Phase 1	Deepana–Pachana	Panchakolasava	25 mL twice daily after food	Agni correction and preparatory therapy
		Vaishwanaram Choornam	Administered with buttermilk	Metabolic regulation
Phase 2	Snehapana	Mahathikthakam Ghritham	Increasing dose from 30 mL to 120 mL	Internal oleation prior to Shodhana
Phase 3	Abhyanga	Mahathikthakam Ghritham mixed with Lanthapala Kera Tailam	During inpatient phase	External oleation
	Swedana	Aragvadhadi Gana	During inpatient phase	Sudation therapy
Phase 4	Virechana	Avipathy Choornam	30 g administered in morning; 6–8 vegas observed	Principal Shodhana procedure
Phase 5	Takradhara	Aragvadhadi Gana processed medicated buttermilk	7 days	Stress reduction and post-procedure stabilization
	Thalam	Ksheerabala Tailam with Kachooradi Choornam	During Takradhara period	Psychological calming and relaxation
Discharge medication	Internal medication	Gugguluthikthakam Ghritham	1 tablespoon daily	Maintenance therapy
	Internal medication	Kaishora Guggulu Gulika	2-0-2	Long-term inflammatory regulation
	External application	Lanthapala Kera Tailam	Local application	Maintenance of remission and skin care

## 3 Outcomes and follow-up

Marked clinical improvement was observed following completion of the first treatment schedule in 2024. Progressive reduction in erythema, scaling, itching, and oozing was noted during treatment, particularly after Snehapana and Vamana. At one-month follow-up, near-complete resolution of psoriatic lesions was achieved, with restoration of near-normal skin appearance and resolution of post-inflammatory hypopigmented lesions over the neck and face.

**Figure 4:**
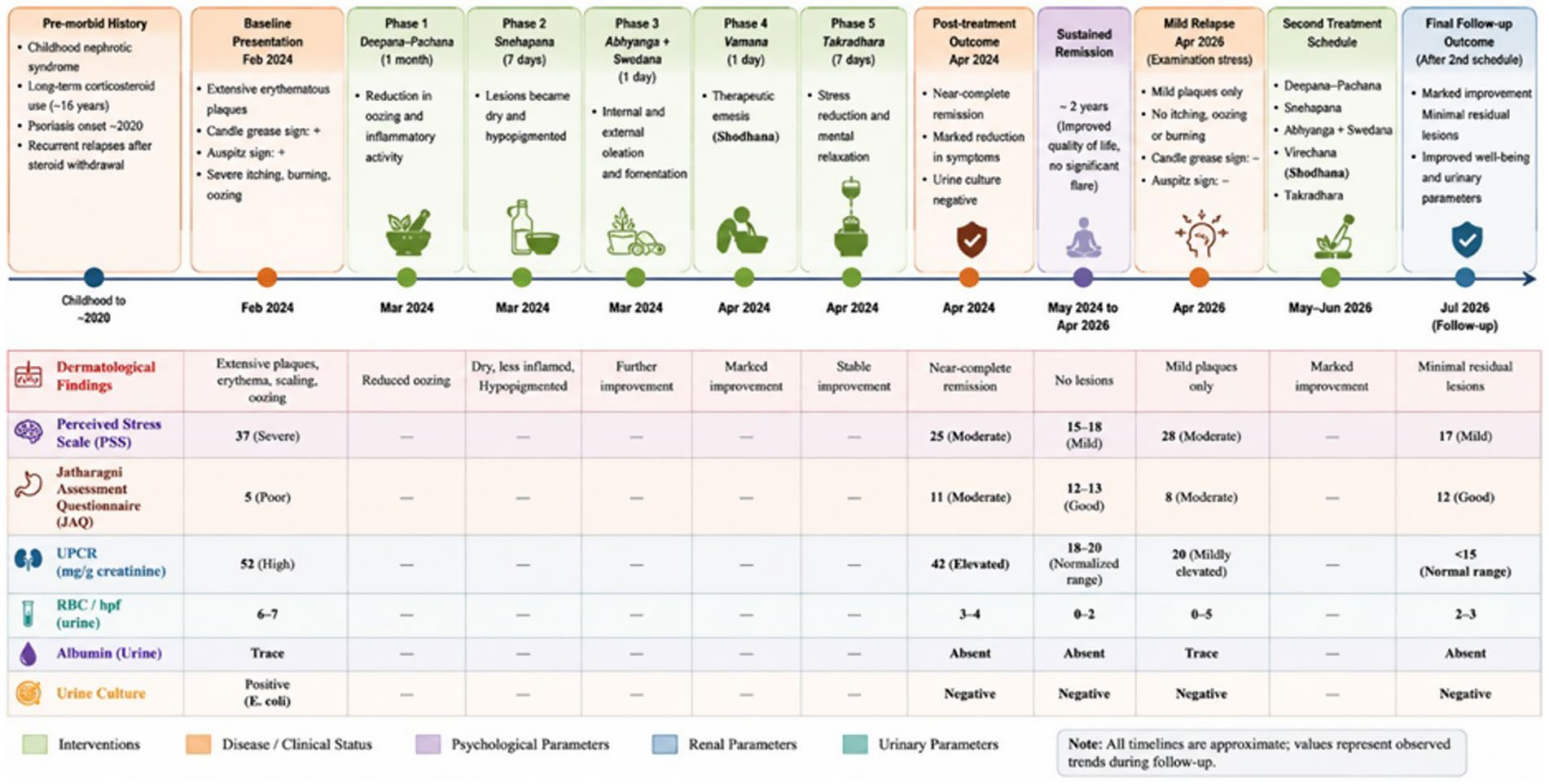
Timeline of clinical course, interventions and outcomes.

The patient subsequently remained in sustained remission for approximately 2 years without significant recurrence. During this period, no major flare episodes were reported. Recurrence occurred in April 2026 and was temporally associated with increased academic stress related to examinations (Table 3). Compared to the initial presentation, the relapse was considerably milder, consisting only of a few small lesions over both lower limbs and a minimal lesion over the right elbow without associated itching, burning sensation, or oozing. Classical dermatological signs such as candle grease sign and Auspitz sign, which had been positive during the initial presentation, were absent during recurrence, suggesting reduced inflammatory severity (Table 4). PASI score reduced markedly from 52 at baseline to 6 following the first treatment schedule. During recurrence in 2026, PASI score was 12 and subsequently reduced to 5 after the second treatment schedule (Figure 4).

**Table 3:** Timeline of major clinical events.

Timepoint	Clinical event
Childhood	Diagnosed with nephrotic syndrome
Childhood–2020	Long-term corticosteroid exposure (~16 years)
2020	Onset of chronic plaque psoriasis
February 2024	Severe flare with extensive plaques, urinary abnormalities, and high stress burden
February–April 2024	First treatment schedule including Deepana–Pachana, Snehapana, Vamana, and Takradhara
April 2024	Marked clinical improvement and near-complete remission
2024–2026	Sustained remission for approximately 2 years
April 2026	Mild recurrence associated with academic stress
April 2026	Second treatment schedule including Deepana–Pachana, Snehapana, Virechana, and Takradhara
Follow-up	Improvement in PASI, stress scores, and urinary parameters

**Table 4:** Longitudinal out comes and follow up.

**Parameter**	Baseline (2024)	After First Treatment (2024)	Recurrence (2026)	After Second Treatment (2026)
Clinical severity	Extensive erythematous plaques with severe itching, burning, scaling, and oozing	Near-complete remission with minimal residual hypopigmentation	Mild recurrence with few small plaques and minimal symptoms	Marked improvement with minimal residual marks
PASI score	52	6	12	5
Candle grease sign	Positive	Negative	Negative	Negative
Auspitz sign	Positive	Negative	Negative	Negative
UPCR	52	42	—	20
Urine RBC/hpf	6–7	3–4	—	2–3
Albuminuria	Trace albumin present	Absent	Absent	Absent
Urine culture	*Enterococcus faecalis* positive	Negative	Negative	Negative
PSS score	37	25	28	17
JAQ score	5	11	8	12
Renal function	Normal creatinine and eGFR	Normal	Normal	Normal
Remission status	Active disease	Sustained remission achieved	Mild relapse after ~2 years	Clinical remission re-achieved

Following the second treatment schedule in 2026, rapid improvement of lesions was again observed with healing of plaques and only minimal residual marks remaining.

Parallel improvement was also observed in psychological stress scores, digestive-metabolic assessment, and urinary parameters over the course of treatment and follow-up. Perceived Stress Scale (PSS) scores reduced after both treatment schedules, while Jatharagni Assessment Questionnaire (JAQ) scores demonstrated progressive improvement in Agni status. Similarly, serial laboratory investigations demonstrated gradual reduction in urine protein–creatinine ratio (UPCR) and improvement in urinary findings over time.

## 4 Discussion

### 4.1 Psoriasis as a systemic inflammatory disorder

Psoriasis is increasingly recognized as a chronic systemic inflammatory disorder rather than a disease limited to the skin (13). Dysregulation of immune pathways involving Th1/Th17 cells and inflammatory cytokines contributes not only to keratinocyte hyperproliferation but also to persistent systemic inflammation (14). Emerging evidence from immunogenetic and molecular studies further supports the concept of psoriasis as a biologically heterogeneous disease involving interconnected inflammatory, metabolic, and immune pathways.

In the present case, the patient exhibited severe chronic plaque psoriasis associated with significant psychosocial burden, recurrent disease activity, and persistent urinary abnormalities. The parallel improvement observed in dermatological manifestations, stress burden, digestive-metabolic assessment, and urinary findings following treatment suggests possible modulation of interconnected systemic inflammatory pathways, rather than isolated cutaneous improvement.

### 4.2 Possible renal involvement in psoriasis

Renal involvement in psoriasis has gained increasing attention in recent years, with studies demonstrating both overt and subclinical renal abnormalities in psoriatic patients. Persistent systemic inflammation, endothelial dysfunction, immune dysregulation, and cytokine-mediated injury have been proposed as possible mechanisms linking psoriasis with renal pathology (15). Associations between psoriasis and IgA nephropathy have also been reported, and recent genetic and transcriptomic analyses suggest possible shared inflammatory pathways between the two conditions (4, 5).

In the present case, elevated urine protein–creatinine ratio (UPCR), persistent microscopic hematuria, and trace albuminuria were observed despite preserved renal function and absence of clinically evident nephrotic syndrome. The gradual reduction in UPCR values and improvement in urinary findings during follow-up may reflect attenuation of systemic inflammatory burden accompanying clinical remission. However, definitive characterization of renal pathology was not possible because renal biopsy and advanced immunological investigations were not performed. Therefore, the renal findings in this patient are best interpreted as possible inflammatory renal involvement associated with systemic psoriatic disease rather than confirmed nephropathy.

### 4.3 Stress and psychoneuroimmunological mechanisms

Psychological stress is a well-recognized trigger for psoriasis onset and exacerbation and may influence disease activity through psychoneuroimmunological mechanisms involving neuroendocrine and inflammatory pathways (6, 7). Stress-related activation of inflammatory signaling may contribute to increased cytokine production, immune dysregulation, and worsening of cutaneous inflammation.

The present patient demonstrated marked psychosocial burden, social withdrawal, and elevated baseline Perceived Stress Scale (PSS) scores. Disease recurrence after approximately 2 years of remission was temporally associated with examination-related stress, further supporting the role of psychological factors in disease activity. Improvement in PSS scores following treatment, together with reduction in clinical severity, may indicate modulation of stress-associated inflammatory pathways. From an Ayurvedic perspective, *Takradhara* and *Thalam* are traditionally considered to possess Pitta-pacifying and calming properties and are commonly employed in conditions associated with psychological stress and heightened inflammatory states. In the present case, improvement in perceived stress scores following these interventions may suggest a possible role in psychophysiological stabilization. The incorporation of Takradhara during both treatment schedules may also have contributed to stress reduction and neurophysiological stabilization, although objective neuroendocrine markers were not assessed (16).

### 4.4 Agni impairment and systemic inflammatory regulation

From an Ayurvedic perspective, psoriasis-like disorders are understood as conditions involving derangement of Doshas especially kapha-pitha, associated with impairment of Agni, broadly representing digestive-metabolic regulatory function. Disturbance of Agni is considered to contribute to systemic imbalance and pathological tissue involvement.

Emerging evidence from microbiome and metabolomic studies increasingly supports the role of metabolic and microbial dysregulation in psoriasis pathogenesis (16). Interactions between the microbiome, immune system, and inflammatory signaling pathways may influence systemic disease activity and chronic inflammation. In the present case, improvement in Jatharagni Assessment Questionnaire (JAQ) scores occurred alongside clinical remission and improvement in urinary parameters. Although direct equivalence between Agni and modern metabolic concepts cannot be established, the observed parallel changes may suggest a relationship between metabolic regulation and inflammatory disease activity (12).

### 4.5 Possible role of staged Shodhana intervention

An important feature of the present case was the staged and individualized therapeutic approach. Rather than proceeding directly to purification procedures, initial management focused on Deepana–Pachana therapy aimed at improving digestive-metabolic function before Shodhana. Clinical improvement during this preparatory phase, including reduction in oozing and inflammatory activity, was observed before initiation of Snehapana and subsequent purification procedures.

Panchakarma-based interventions are traditionally utilized in Ayurveda as staged purification procedures intended to restore systemic physiological balance and reduce disease burden (17). In the present case, the sequential therapeutic approach involving Deepana–Pachana, Snehapana, and Shodhana procedures was associated with progressive reduction in inflammatory severity and sustained remission during follow-up. The repeated clinical response observed following both treatment schedules suggests a possible role of Shodhana-based interventions in modulating disease activity beyond temporary symptomatic suppression. During the initial presentation, classical inflammatory signs such as candle grease sign and Auspitz sign were clearly positive, whereas these signs were absent during the milder recurrence after 2 years. Substantial reduction in PASI scores observed following both treatment schedules further supported the marked clinical improvement documented through serial photographic assessment. This difference in recurrence phenotype, together with sustained remission and reduced symptom severity, may indicate alteration in disease behavior over time. However, the precise mechanisms underlying these observations remain uncertain and require further systematic investigation.

The staged therapeutic approach employed in this case was designed to address different phases of disease expression. Deepana–Pachana was administered initially to improve digestive-metabolic function and reduce symptom burden, which was clinically reflected by reduction in pruritus and burning sensation. Snehapana and subsequent Shodhana procedures were followed by progressive reduction in PASI score and inflammatory severity. Although the precise biological mechanisms remain uncertain, the observed improvements in dermatological severity, perceived stress, and urinary inflammatory parameters suggest a possible systemic effect extending beyond local skin manifestations.

### 4.6 Clinical significance of prolonged remission

One of the clinically significant observations in the present case was the maintenance of near-complete remission for approximately 2 years following the first treatment schedule. Recurrence, when it occurred, was considerably milder than the initial disease presentation and lacked severe inflammatory features such as intense itching, burning, oozing, and positive Auspitz sign.

This prolonged remission period is noteworthy in the context of chronic relapsing psoriasis with prior dependence on prolonged corticosteroid therapy. The findings raise the possibility that the integrative therapeutic approach may have influenced broader systemic inflammatory and psychometabolic pathways associated with disease recurrence. Although conclusions regarding long-term disease modification cannot be established from a single case, the longitudinal follow-up provides clinically relevant observations warranting further study.

### 4.7 Limitations

This report represents a single observational case and therefore findings cannot be generalized. Although PASI scores were documented, additional standardized dermatology quality-of-life indices such as DLQI were not recorded. Renal biopsy, cytokine profiling, microbiome analysis, and advanced immunological investigations were not performed, limiting definitive characterization of renal pathology and mechanistic interpretation of treatment response.

In addition, the patient received antimicrobial formulations in Ayurveda during the initial treatment phase because of positive urine culture findings, which may have contributed to improvement in urinary abnormalities. The independent contribution of individual therapeutic components, including Deepana–Pachana, Shodhana procedures, Takradhara, and follow-up medications, cannot be isolated. Further controlled studies integrating clinical, biochemical, immunological, and psychometric parameters are required to validate these observations.

### 4.8 Ethics statement

Written informed consent was obtained from the patient for publication of this case report and accompanying clinical photographs. All efforts were made to maintain patient confidentiality and anonymity throughout the manuscript, and no identifiable personal information has been disclosed. As this manuscript describes a single-patient case report and does not involve a prospective interventional research study, formal institutional ethics committee approval was not obtained.

### 4.9 Patient perspective

The patient reported that the skin lesions had significantly affected her confidence, social interactions, and emotional well-being prior to treatment. She described experiencing embarrassment and social withdrawal because of the visible nature of the disease and recurrent flare episodes. Following treatment, the patient reported substantial improvement in both physical symptoms and psychological well-being, particularly due to reduction in itching, visible lesions, and recurrence severity. The prolonged remission period also improved her confidence in managing daily academic and social activities.

## 5 Conclusion

This case demonstrates the potential role of a staged Ayurvedic intervention in achieving sustained clinical remission and reducing disease severity in chronic plaque psoriasis associated with persistent urinary abnormalities and significant psychological stress. Parallel improvement observed in dermatological manifestations, stress scores, digestive-metabolic assessment, and urinary parameters suggests a possible integrative systemic effect involving interconnected inflammatory, psychoneuroimmunological, and metabolic pathways.

The prolonged remission period and milder recurrence pattern observed during follow-up further highlight the potential value of individualized and systems-oriented management approaches in chronic inflammatory disease. However, as this report represents a single observational case without advanced immunological or histopathological evaluation, definitive mechanistic conclusions cannot be established. Further large-scale longitudinal studies integrating clinical, biochemical, immunological, microbiome, and psychometric assessments are required to validate these findings and better understand the potential role of integrative therapeutic approaches in psoriasis management.

## Data Availability

The original contributions presented in the study are included in the article/supplementary material, further inquiries can be directed to the corresponding author/s.
